# Chikungunya as a paradigm for emerging viral diseases: Evaluating disease impact and hurdles to vaccine development

**DOI:** 10.1371/journal.pntd.0006919

**Published:** 2019-01-17

**Authors:** Giovanni Rezza, Scott C. Weaver

**Affiliations:** 1 Department of Infectious Diseases, Istituto Superiore di Sanità, Rome, Italy; 2 Institute for Human Infections and Immunity and Department of Microbiology and Immunology, University of Texas Medical Branch, Galveston, Texas, United States of America; George Washington University School of Medicine and Health Sciences, UNITED STATES

## Abstract

Chikungunya fever (CHIKF) is an emerging infectious disease caused by an alphavirus transmitted by *Aedes* spp. mosquitoes. Because mosquito control programs are not highly efficient for outbreak containment, vaccines are essential to reduce the burden of disease. Although no licensed vaccine against CHIKF is yet available, many highly promising candidates are undergoing preclinical studies, and a few of them have been tested in human trials of phase 1 or 2. Here, we review recent findings regarding the need for a CHIKF vaccine and provide an update on vaccines nearing or having entered clinical trials. We also address needs to tackle bottlenecks to vaccine development—including scientific and financial barriers—and to accelerate the development of vaccines; several actions should be taken: (i) design efficacy trials to be conducted during the course of outbreaks; (ii) evaluate the opportunity for adopting the “animal rule”for demonstration of efficacy for regulatory purposes; (iii) strengthen the collective commitment of nations, international organizations, potential donors and industry; (iv) stimulate public and/or private partnerships to invest in vaccine development and licensure; and (v) identify potential markets for an effective and safe CHIKF vaccine.

Chikungunya virus (CHIKV) is an RNA alphavirus belonging to the *Togaviridae* family, first identified in Tanzania in 1952. During epidemics and endemic circulation, CHIKV is transmitted by *Aedes aegypti* and, to a lesser extent, by *A*. *albopictus* mosquitoes. Infection with CHIKV typically causes a self-limiting febrile illness, chikungunya fever (CHIKF), characterized by chronic, severe joint pain, and sometimes accompanied by an itchy maculo-papular skin rash. Severe complications, such as encephalitis, may occur in the elderly and in individuals with comorbidities, and peripartum infections can be fatal or involve severe neurologic sequelae in fetuses and infants [1, 2]. CHIKV is enzootic in Africa, where transmission involves different arboreal *Aedes* spp. vectors and nonhuman primates (NHP) in forested habitats. Direct spillover infections of humans from these enzootic cycles probably occur in many regions of Sub-Saharan Africa. In Asia, CHIKV is endemic and causes recurrent and sometimes large epidemics, especially in the Indian subcontinent and in Southeast Asia [1–3]. In 2004, CHIKV reemerged to cause large outbreaks, which began on the coast of Kenya and ravaged several Indian Ocean islands and the Indian subcontinent in the years 2005 to 2006, before spreading to initiate transmission in Southeast Asia [4, 5]. A few years later, CHIKF outbreaks were also reported in the Arabian Peninsula [6]. In 2013, autochthonous (locally originating) chains of transmission of CHIKV were identified for the first time in the Americas [7]. CHIKV has expanded its range of activity to include temperate regions in part by adapting for transmission by *A*. *albopictus*, including two outbreaks in Italy in 2007 and 2017 [8, 9] and two in France. The unabated spread and increasing burden of CHIKF underscores the need to develop an effective vaccine [10].

Vaccines for CHIKF have been developed for several decades, and comprehensive reviews of these have been published in recent years [11–13]. Here, we focus on recent findings regarding the need for a CHIKF vaccine. We also provide an update on CHIKF vaccines nearing or having entered clinical trials, and address bottlenecks to further development, including scientific and financial barriers.

## Understanding the impact of CHIKF

**Fig 1:**
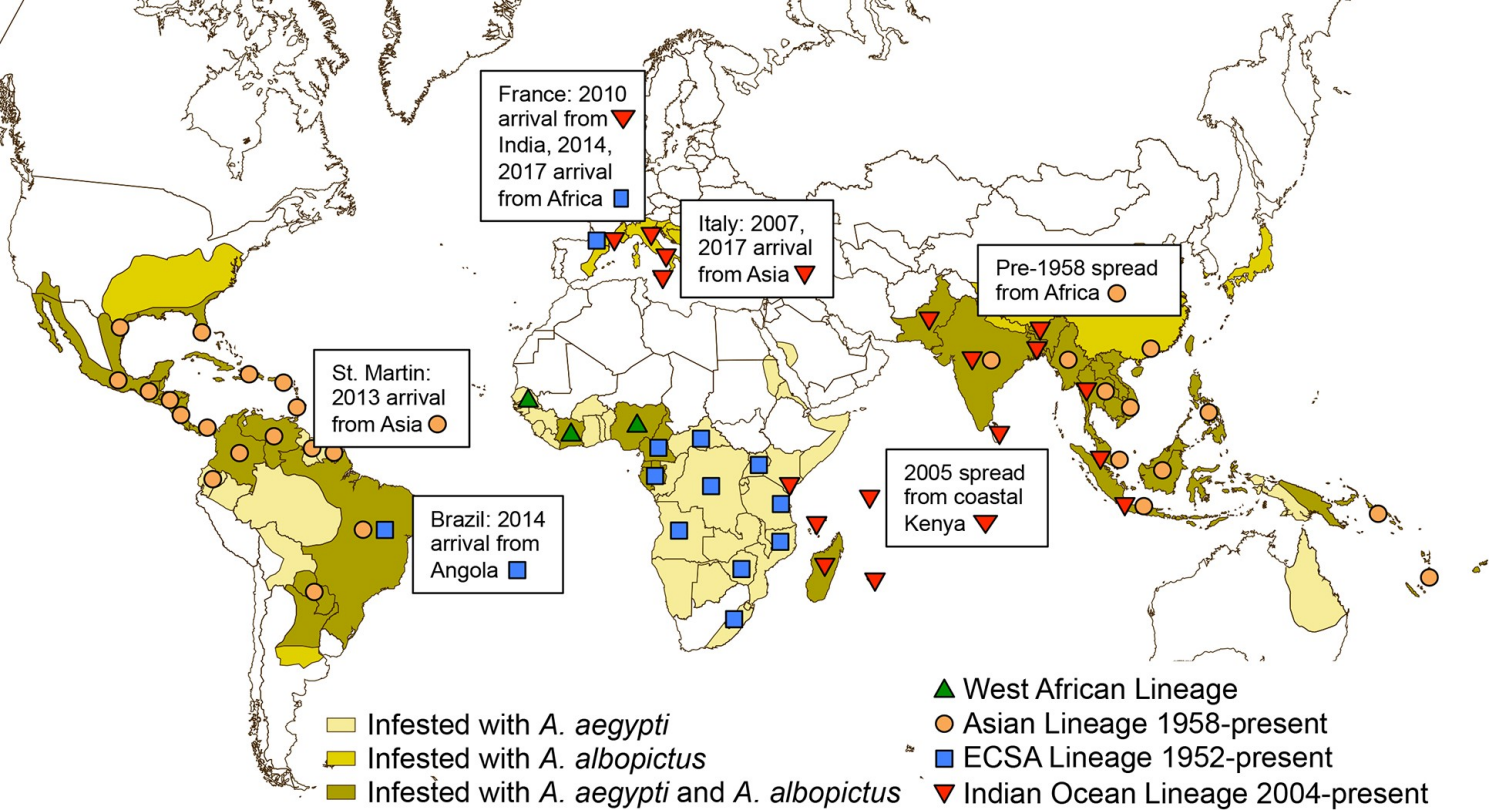
World map with countries where autochthonous (locally initiated) chains of CHIKV transmission have been identified. Data from World Health Organization (http://www.who.int/emergencies/diseases/chikungunya/en/) and Pan American Health Organization (https://www.paho.org/hq/index.php?option=com_topics&view=article&id=343&Itemid=40931&lang=en). CHIKV, chikungunya virus.

Developing a safe and effective vaccine against CHIKF—as well as against other reemerging diseases, such as Ebola, Lassa, or Nipah—is important for several reasons. The impact of CHIKF in terms of burden of disease, work and school absenteeism, and other financial costs is particularly high, especially given its formidable epidemic potential. A paradigmatic example is provided by the 2006 epidemic wave that occurred in India, in which more than 1,500,000 cases of CHIKF were reported (http://www.who.int/denguecontrol/arbo-viral/other_arboviral_chikungunya/en/). Furthermore, the global impact of CHIKV is constantly growing, due to the introduction and spread of the virus into new continents in which it finds optimal conditions for its expansion, including, in some cases, a completely naïve population (Fig 1). The capacity of CHIKV to adapt to a new mosquito vector has been demonstrated during the Indian Ocean epidemic, when a series of mutations increased fitness for transmission by *A*. *albopictus* [14, 15], a mosquito that can survive at higher latitudes than *A*. *aegypti* [16]. This may lead to the occurrence of outbreaks in temperate climates, as seen in Europe [8, 9].

Secondly, although the case–fatality rate of CHIKF is relatively low—usually well below 1% [1, 2]—it may be underestimated in small outbreaks and in epidemic waves that occur in resource-poor countries. Excess mortality was investigated during an outbreak in Mauritius, and the case–fatality rate was estimated to be around 2.3 per 1000 [17]. Although “Old World” alphaviruses are not considered neurotropic sensu strictu [18], cases of meningoencephalitis have been documented, especially during Indian outbreaks [1], and fatal encephalitis has been reported with CHIKV infection both in Italy [8] and in La Reunion, where it was observed in patients mostly below 1 year or over 65 years of age [19]. Relatively high case–fatality rates (17%) and persistent disability (30% to 45%) were documented among patients with CHIKV-associated encephalitis. An increased incidence of other neurological syndromes, such as Guillain-Barré syndrome, was also reported during a CHIKF outbreak in French Polynesia [20]. These findings provide evidence of severe disease associated with CHIKV infection, which may have a high impact in terms of hospitalization and mortality during large outbreaks.

Thirdly, persistent arthralgia and joint swelling are common long-term manifestations of CHIKF. Unlike other mosquito-borne viruses such as dengue, Zika, and even yellow fever, CHIKV typically causes symptomatic infection, and consequently outbreaks are accompanied by high attack rates. Chronic joint pain, along with asthenia and mood changes, is a common cause of quality-of-life impairment. Ninety-four percent of symptomatic travelers infected in La Reunion complained of joint or bone pain 6 months after the epidemic peak; this pain was continuous in 41% of the cases. The effect of chronic symptoms on the quality of life was defined as totally disabling or important in almost half of the patients, whereas only 16% reported a normal mood [21]. A study conducted in La Reunion on 147 individuals over the age of 16 found that 84 confirmed cases (57%) self-reported rheumatic symptoms; of these, 63% reported permanent pain, whereas 37% had recurrent symptoms. An age of over 45 years (odds ratio [OR] 3.9), severe initial joint pain (OR 4.8), and the presence of underlying osteoarthritic comorbidity (OR 2.9) were independent predictors of nonrecovery [22]. An assessment of 173 individuals with CHIKF conducted in Mauritius found that 79% reported persisting musculoskeletal symptoms 27.5 months after infection, associated with older age, female gender, and a baseline symmetrical distribution of arthralgia [23]. A study conducted on Italian patients found that over 66% of those with CHIKF developed long-lasting rheumatic disorders, leading to functional impairment affecting daily living activities up to one year after infection [24]. Therefore, the long-term impact of CHIKF is far from negligible in terms of suffering, need for care, impairment of work ability, psychological problems, diminished quality of life, and associated economic costs.

Unfortunately, little information is available on the economic costs of CHIKV infection. Healthcare costs during the La Reunion in 2005 to 2006 epidemic were estimated: the medical management of CHIKF was associated with a major economic burden, with 60% of the CHIKF-related expenditure attributable to direct medical costs, such as medical consultation (47%), hospitalization (32%), and drug consumption (19%). The cost of analgesics accounted for 80% of the CHIKF-related drug expenditures. Loss of productivity, measured as absenteeism costs, was also high [25]. Studies conducted in 2006 in Gujarat, India, estimated an immediate cost to household of CHIKF and dengue around 3.8 billion Indian rupees (ca. US$55 million) per annum, whereas another study conducted in Ahmedabad, a city of 3.5 million people in the State of Gujarat, found that the disease affected primarily working-age adults, with an immediate cost of the outbreak due to lost wages and treatment of approximately US$1.7 million based only on officially reported cases; these figures are probably underestimated, because only 23% of the cases seek treatment within public facilities [26]. Another study estimated a total of about 40 million cases in the Americas, with a burden of 24 million disability life years (DALYs) lost and about US$185 billion in societal cost [27]. Therefore, although CHIKF is in most cases a relatively mild illness, its burden during epidemic waves may be impressive. Finally, when CHIKF outbreaks occur in tourist destinations, economic loss due to decreased numbers of visitors may cause further economic impacts. The under-recognition of the potential impact of CHIKF led to undesired political consequences during the epidemic waves on the tourist destination island of La Reunion, with the French government being accused of negligence and delays in outbreak response [65].

Therefore, for the reasons reported above, the development of a safe and effective vaccine against CHIKF would have a significant impact on the global burden of this disease with important health, economic, and ethical implications: (i) because of the high infection rates during epidemic waves, the disease burden may be relatively high. An effective vaccine would reduce the number of cases and hospitalizations worldwide, producing economic benefits through the reduction of absenteeism, lower costs for care and hospitalizations, and reduced loss of income associated with tourism; (ii) countries outside the tropics might also benefit from the development of a CHIKF vaccine, because the virus may be introduced from endemic and/or epidemic areas or enzootic circulation and eventually spread to regions where competent urban vectors are present; (iii) protection of travelers and military personnel may be another positive outcome of vaccine development; (iv) there are clear ethical implications in the development of a vaccine against an emerging neglected disease that primarily affects resource-limited parts of the globe.

## CHIKF vaccine candidates: State of development

Although most research on vaccines against CHIKF began only during the past 10 years, several inactivated and attenuated vaccine candidates and newer platforms have been tested in preclinical and human trials, showing promising results. Here, we focus mainly on vaccines that have undergone extensive preclinical testing, including in nonhuman primates (NHPs), and/or have entered clinical trials.

### Formalin-inactivated vaccines

Initially developed in the 1970s, these induce neutralizing antibodies in animal models and humans [28, 29]. However, most development of these vaccines was discontinued decades ago because of high-production costs and risks associated with handling large quantities of virulent, wild-type, virus prior to inactivation. Therefore, although newer inactivated candidates have been tested with promising results [30, 31], most subsequent vaccine research was directed towards live-attenuated candidates.

### Live-attenuated vaccines

A serially passaged, empirically live-attenuated candidate vaccine called strain 181/clone25 [32] was eventually evaluated in a Phase 2 clinical study [33]: all vaccinated individuals developed neutralizing antibodies but 8% of them experienced mild, transient joint pain, a common symptom of CHIKF. The occurrence of arthralgia suggested insufficient and/or unstable attenuation; later studies indicated that the 181/clone25 attenuation is mediated by only two point mutations, which can revert following vaccination [34]. In other studies, seroconversion rates were low among those who had been previously vaccinated with other live-attenuated alphavirus vaccines (i.e., Venezuelan equine encephalitis virus), suggesting immunological interference [35]. Ultimately, development of this live-attenuated vaccine was discontinued not only because of reactogenicity and the absence of widespread and well-documented outbreaks but also due to the scarcity of funding and concerns regarding marketing [36, 37].

To design better live-attenuated candidates, genetically engineered complementary DNA (cDNA) clone-based, rationally attenuated CHIKV strains were more recently developed and successfully tested in animal models [38, 39], including NHP [40]. These vaccines contain very specific and stable mutations, providing better safety profiles compared with earlier live-attenuated vaccines such as 181/clone25, and retaining strong immunogenicity [12].

The first of these rationally attenuated CHIKF vaccines to undergo extensive preclinical testing employed a picornavirus-derived internal ribosome entry site to replace the CHIKV subgenomic promoter [41]. This replacement is highly stable after serial cell culture and mouse brain passages [42], and a single dose of the vaccine elicits robust neutralizing antibody responses and protects mice and NHPs against virulent CHIKV challenge, including weight change, fever, and viremia in cynomolgus macaques [40]. Protection after a single dose in mice lasts over 9 months, and protection of both mice and NHPs from this Indian Ocean lineage-derived strain also extends to Asian/American lineage CHIKV strains [43].

Other clone-derived, rationally attenuated CHIKF vaccine candidates include strains with deletions of a large part of the nonstructural protein 3 (nsP3) gene or the entire 6K/TF gene [38]. These vaccines, either administered as viral particles or infectious genomes launched from DNA, are highly immunogenic and efficacious in mice [38]. The nsP3 deletion vaccine, Δ5nsP3 (VLA1553), is now in the recruitment stage for a Phase 1 clinical trial (Table 1).

**Table 1:** CHIKF vaccines in late preclinical or clinical development.

Vaccine name	Developer	Vaccine type	Doses required/tested	Current stage of testing (clinicaltrials.gov identifier)	References
TSI-GSD-218 (181/clone25)	United States Army Medical Research Institute of Infectious Diseases, University of Maryland	Live-attenuated CHIKV strain	1	Completed Phase 2	[32, 33]
VRC-CHKVLP059-00-VP (PXVX0317 CHIKV-VLP)	US National Institutes of Health, PaxVax	Virus-like particle assembled from CHIKV proteins expressed in mammalian cells	2	Phase 2 (NCT02562482, NCT01489358, NCT03483961)	[49, 50]
MV-CHIK	Inst. Pasteur, Themis Bioscience	Recombinant live- attenuated measles vaccine expressing CHIKV virus-like particles derived from the structural protein genes	2	Phase 2 (NCT03101111, NCT02861586)	[44, 63]
VLA1553	Valneva, Austria	Recombinant CHIKV with nsP3 deletion	1	Recruiting for Phase 1 (NCT03382964)	[38, 64]
VAL-181388	Moderna Therapeutics	mRNA encoding the CHIKV structural proteins	Not reported	Recruiting for Phase 1 (NCT03325075)	
pMCE321	University of Pennsylvania, Philadelphia	Structural protein genes E3, E2, and E1 linked in a single DNA construct with furin cleavage sites between them	3	Late preclinical (immunogenicity and efficacy in mice, immunogenicity in NHPs)	[58]
CHIKV/IRES	University of Texas Medical Branch, Takeda Pharmaceuticals	Recombinant CHIKV with subgenomic promoter replaced by internal ribosome entry site to down-regulate structural proteins and prevent mosquito infection	1	Late preclinical (immunogenicity and efficacy in mice, NHPs)	[41–43]
EILV/CHIKV	University of Texas Medical Branch	Recombinant Eilat/chikungunya chimeric virus that is replication-defective in vertebrate cells	1	Late preclinical (immunogenicity and efficacy in mice, NHPs)	[48]

**Abbreviations:** CHIKF, chikungunya fever; CHIKV, chikungunya virus; EILV, Eilat virus; NHP, nonhuman primate; nsP3, nonstructural protein 3.

### Viral-vectored vaccines

A CHIKF vaccine in advanced stages of clinical development employs an attenuated measles virus strain as a vector to express the CHIKV structural proteins [44]. In a Phase 1 trial, this vaccine was well tolerated and induced neutralizing antibodies in 44% of volunteers receiving a single low-dose, 92% receiving a medium-dose group, and 90% receiving a high-dose. A booster raised seroconversion to 100%, and immunogenicity was not affected by preexisting anti-measles immunity. This vaccine is now in Phase 2 trials (Table 1).

Another unique viral-vectored vaccine, EILV/CHIKV, was developed based on the insect-specific alphavirus, Eilat (EILV), which is completely defective for replication in vertebrate cells [45, 46]. When chimerized to replace the Eilat structural polyprotein open reading frame (ORF) with that of CHIKV, the host restriction remains, as indicated by a complete lack of RNA genome replication and virus production in several vertebrate cell lines as well as in serial passages in infant, immunodeficient mouse brains, the most permissive vertebrate environment for most alphaviruses [47, 48]. A single dose of this replication-deficient vaccine produced in mosquito cells protects mice and NHPs from all measures of disease and viremia, with murine protection extending beyond 9 months. This remarkable immunogenicity (single dose of a replication-defective vaccine) is mediated, at least in part, but the normal endocytic pathway of the vaccine particles, which are structurally identical to wild-type CHIKV.

### Virus-like particle vaccines

In addition to inactivated and viral vectored CHIKF vaccines, virus-like particles (VLPs) have also been developed and advanced to clinical trials. Expression via electroporation of mammalian cells of the CHIKV structural polyprotein ORF in a DNA plasmid form produces VLPs with identical protein structure to CHIKV. These VLPs elicit neutralizing antibodies against envelope proteins from diverse CHIKV strains, and immunized NHPs produce high-titer neutralizing antibodies that passively protect immunodeficient mice against lethal infection [49]. In a Phase 1 trial, this VLP vaccine was well tolerated and induced neutralizing antibodies in all dose groups after two vaccinations; a significant boost occurred after a third vaccination [50]. This vaccine is now in Phase 2 clinical trials (Table 1).

### Nucleic acid-based vaccines

Finally, nucleic acid-based vaccines against CHIKV are under development. These vaccines have clear advantages, from ease of production, to safety, to the ability to induce both humoral and cell-mediated immunity; however many have shown a relatively low immunogenicity, requiring large doses, repeated boosters, and the use of adjuvants [12]. One RNA-based vaccine, VAL-181388, is recruiting for a Phase 1 clinical trial (Table 1).

Overall, nearly 30 vaccine candidates against CHIKF have been reported, but only four of these have entered Phase 1 or 2 trials [51–53] (Table 1). Whether any of these vaccine candidates will advance to Phase 3 trials cannot be easily predicted and will be based on the perceived market as well as the identification of a suitable site with adequate incidence of CHIKF to demonstrate efficacy.

### Bottlenecks for chikungunya vaccine development: Which obstacles should be removed?

CHIKF outbreak control is hampered by the lack of licensed vaccines that can be used in preventive immunization programs and for emergency response. Although the technical challenges of developing a CHIKF vaccine are not as great as those for some other viral diseases such as dengue, in which multiple serotypes must be targeted and partial immunity can lead to disease enhancement [54], there are several critical obstacles and financial constraints that need to be overcome in order to make available an affordable and effective vaccine.

First of all, barriers to the acquisition of human efficacy data for vaccine candidates due to the unpredictable nature of CHIKF epidemics may delay the development of a vaccine, even though promising candidates are available. For example, randomized controlled trials, which are considered the gold-standard for evaluating vaccine efficacy, may not be feasible during interepidemics periods because of the low expected number of cases; for this reason, epidemic events with a large number of cases may represent unique opportunities to ensure study power for testing vaccine candidates in efficacy trials. Approaches to overcoming these barriers include the development of platform technologies in which the critical antigens of a newly emerging viral strain can be rapidly incorporated into DNA or RNA platforms with proven safety records. However, based on initial results with DNA Zika vaccines that were generated within a few months of the recognition of congenital Zika Syndrome, these vaccines require multiple doses and immunity is not long lived; they are therefore far from ideal for intervening during an explosive epidemic or for long-term protection in endemic locations. Viral-vectored platforms such as vesicular stomatitis or measles can also be rapidly adapted for new viral targets and may offer more rapid and durable immunity.

Another approach to overcoming challenges of unpredictably emerging viral diseases is acceleration of clinical testing of new vaccine candidates and providing a robust rationale for particular trial designs and regulatory pathways. Therefore, vaccine trials should be designed very carefully to implement quickly and maximize their results during outbreaks. Potential opportunities for CHIKF vaccine testing may include cities with histories of recurrent dengue outbreaks (dengue and CHIKV share the same human-amplified transmission cycles, so regions susceptible to one should eventually have outbreaks of the other); for example, Sao Paulo State, Brazil, and Iquitos, Peru, have still not experienced major CHIKF outbreaks. However, ethical concerns with placebo-immunizing at-risk populations during an epidemic may need to be overcome with nontraditional designs, such as that used during the Ebola vaccine trials in West Africa [55].

There may also be opportunities to perform efficacy trials in regions endemic for CHIKF, but the typical misdiagnosis of CHIKF as dengue fever [56] will need to be overcome with improved surveillance and diagnostics to identify such opportunities. Finally, CHIKF will continue to occur mainly in poor-resource countries located in tropical areas, where the presence of trained and well-equipped clinical sites, which are essential for the implementation of clinical trials, can be challenging. However, there may be opportunities to capitalize on sites already developed for dengue vaccine trials, which are generally in locations endemic for both viruses.

In case these challenges to clinical efficacy trials cannot be overcome, alternative strategies should be considered. For example, it will be important to obtain reliable information on correlates of immune protection, which are essential in order to apply the so-called “animal rule.” This entails the use of surrogate end-points derived from animal data instead of the results of human trials. This approach could be considered as an alternative option when large efficacy studies on humans, which are usually requested for traditional regulatory approval, are virtually impossible to realize [57]. For example, if human antibodies against CHIKV developed from individuals vaccinated in phase 1 and 2 trials are transferred to NHPs and are demonstrated to confer protection, this could provide surrogate evidence of vaccine efficacy, leading to a provisional license [58]. In this regard, the level of neutralizing antibodies has been already proposed for use as a surrogate marker of vaccine-induced protection [10, 36, 49, 58]. Although there is strong evidence that neutralizing antibodies against CHIKV protect against infection and disease, the lack of compete understanding of chronic arthritis, and its determinants, could limit the ability to relate animal efficacy to human protection. Further work to model arthralgia and arthritis in NHPs could greatly enhance the value of preclinical CHIKF vaccine testing. Other regulatory considerations, including “traditional approval,” “accelerated approval,” and the “animal rule,” have been reviewed extensively in another article focused on CHIKF vaccines [13].

Obstacles to the provision of scientific evidence are not only represented by the barriers described above to vaccine development. In fact, most research and development (R&D) projects do not deliver a licensed vaccine for routine or targeted immunization—not because of methodological problems, but due to political and economic obstacles [59]. In fact, neglected diseases disproportionally affect poor and marginalized populations, and vaccines may have low returns on investment, so commercial firms may be reluctant to commit themselves to the expensive development and licensure of vaccine candidates, which typically totals hundreds-of-millions of US dollars [60]. To overcome this problem, several strategies may be implemented, including the creation of public and/or private partnerships, the identification of target population groups for vaccination to ensure a potential market, such as the military market, travelers and tourists, and the commitment of donor agencies and affected and/or donor countries [61]. Combination private and public consortia should address those vaccine development projects that are not considered highly profitable by industry in the absence of support from the governments of industrial countries. A recent example is the Coalition for Epidemic Preparedness Innovations (CEPI), funded by both government entities and private foundations, and include partners from the pharmaceutical industry, which is funding late preclinical and clinical development of vaccines for infections by Lassa, Nipah, and MERS coronavirus [62].

## Conclusion

In summary, the burden of disease caused by CHIKV is very high, due to the expanding geographic range of virus activity, increasing numbers of cases worldwide, and to the severe and long-lasting arthralgic sequelae of the disease. Developing an effective vaccine is crucial to contain outbreaks and to reduce the clinical and financial impact of CHIKF at the global level. However, as for other neglected and sporadically emerging diseases, barriers to traditional vaccine development and licensure need to be overcome by investing appropriate resources, which may require novel strategies to bring together diverse stakeholders.

**Key learning points**

- The burden of disease due to CHIKV is high due to recurrent epidemics and outbreaks in previously CHIKV-free areas
- CHIKV causes a major public health impact, especially due to persistent joint pain that can be highly debilitating
- Because mosquito control programs against CHIKV vectors have limited efficacy in controlling transmission, an effective vaccine is urgently needed
- Several promising vaccine candidates are currently in Phase 1 and/or 2 clinical trials
- Public and/or private partnerships are needed to incentivize and accelerate vaccine R&D and licensure

**Top five papers**

Venturi G, Di Luca M, Fortuna C, Remoli ME, Riccardo F, Severini F, et al. Detection of a chikungunya outbreak in Central Italy, August to September 2017. Euro Surveill. 2017;22(39). doi: 10.2807/1560-7917.ES.2017.22.39.17-00646. PubMed PMID: 29019306; PubMed Central PMCID: PMCPMC5709953.

Ramsauer K, Schwameis M, Firbas C, Mullner M, Putnak RJ, Thomas SJ, et al. Immunogenicity, safety, and tolerability of a recombinant measles-virus-based chikungunya vaccine: a randomised, double-blind, placebo-controlled, active-comparator, first-in-man trial. The Lancet infectious diseases. 2015. Epub 2015/03/06. doi: 10.1016/S1473-3099(15)70043-5. PubMed PMID: 25739878.

Chang LJ, Dowd KA, Mendoza FH, Saunders JG, Sitar S, Plummer SH, et al. Safety and tolerability of chikungunya virus-like particle vaccine in healthy adults: a phase 1 dose-escalation trial. Lancet. 2014. Epub 2014/08/19. doi: 10.1016/S0140-6736(14)61185-5. PubMed PMID: 25132507.

Weaver SC, Lecuit M. Chikungunya virus and the global spread of a mosquito-borne disease. N Engl J Med. 2015;372(13):1231–9. Epub 2015/03/26. doi: 10.1056/NEJMra1406035. PubMed PMID: 25806915.

Yang S, Fink D, Hulse A, Pratt RD. Regulatory considerations in development of vaccines to prevent disease caused by Chikungunya virus. Vaccine. 2017;35(37):4851–8. doi: 10.1016/j.vaccine.2017.07.065. PubMed PMID: 28760614.

## References

[1] Pialoux G, Gauzere BA, Jaureguiberry S, Strobel M. Chikungunya, an epidemic arbovirosis. The Lancet infect. Dis. 2007;7(5):319–27. 10.1016/S1473-3099(07)70107-X . 17448935

[2] Weaver SC, Lecuit M. Chikungunya virus and the global spread of a mosquito-borne disease. N Engl J Med. 2015;372(13):1231–9. Epub 2015/03/26. 10.1056/NEJMra1406035 . 25806915

[3] Zuckerman AJ, Banatvala JE, Pattison JR, Griffiths PD, Schaub BD. Principle and practice of Clinical Virology, 5th Edition. West Sussex, England: J Wiley & Sons; 2005.

[4] Chikungunya and dengue, south-west Indian Ocean. 2006. WHO Weekly Epidemiol. Record. 2006;81:105–116. 16673456

[5] Charrel RN, de Lamballerie X, Raoult D. Chikungunya outbreaks—the globalization of vectorborne diseases. N Engl J Med. 2007;356(8):769–71. 10.1056/NEJMp078013 . 17314335

[6] Rezza G, El-Sawaf G, Faggioni G, Vescio F, Al Ameri R, De Santis R, et al Co-circulation of Dengue and Chikungunya Viruses, Al Hudaydah, Yemen, 2012. Emerg infect Dis. 2014;20(8):1351–4. 10.3201/eid2008.131615 25061762 PMC4111199

[7] Cassadou S, Boucau S, Petit-Sinturel M, Huc P, Leparc-Goffart I, Ledrans M. Emergence of chikungunya fever on the French side of Saint Martin island, October to December 2013. Euro Surveill. 2014;19(13). . 24721536 10.2807/1560-7917.es2014.19.13.20752

[8] Rezza G, Nicoletti L, Angelini R, Romi R, Finarelli AC, Panning M, et al Infection with chikungunya virus in Italy: an outbreak in a temperate region. Lancet. 2007;370(9602):1840–6. 10.1016/S0140-6736(07)61779-6 . 18061059

[9] Venturi G, Di Luca M, Fortuna C, Remoli ME, Riccardo F, Severini F, et al Detection of a chikungunya outbreak in Central Italy, August to September 2017. Euro Surveill. 2017;22(39). 10.2807/1560-7917.ES.2017.22.39.17–00646 29019306 PMC5709953

[10] Butler D. Health officials push for vaccine against neglected tropical virus. 2018. Available from: http://www.nature.com/articles/d41586-018-01637-7. [cited 2018 Dec 21].

[11] Erasmus JH, Rossi SL, Weaver SC. Development of Vaccines for Chikungunya Fever. J Infect Dis. 2016;214(suppl 5):S488–S96. 10.1093/infdis/jiw271 27920179 PMC5137239

[12] Powers AM. Vaccine and Therapeutic Options To Control Chikungunya Virus. Clinical microbiology reviews. 2018;31(1). 10.1128/CMR.00104-16 . 29237708 PMC5740971

[13] Yang S, Fink D, Hulse A, Pratt RD. Regulatory considerations in development of vaccines to prevent disease caused by Chikungunya virus. Vaccine. 2017;35(37):4851–8. 10.1016/j.vaccine.2017.07.065 . 28760614

[14] Tsetsarkin KA, Chen R, Weaver SC. Interspecies transmission and chikungunya virus emergence. Curr Opin Virol. 2016;16:143–50. 10.1016/j.coviro.2016.02.007 26986235 PMC4824623

[15] Tsetsarkin KA, Chen R, Yun R, Rossi SL, Plante KS, Guerbois M, et al Multi-peaked adaptive landscape for chikungunya virus evolution predicts continued fitness optimization in Aedes albopictus mosquitoes. Nature Comm. 2014;5:4084 10.1038/ncomms5084 . 24933611 PMC7091890

[16] Kraemer MU, Sinka ME, Duda KA, Mylne AQ, Shearer FM, Barker CM, et al The global distribution of the arbovirus vectors *Aedes aegypti* and *Ae. albopictus*. Elife. 2015;4 10.7554/eLife.08347 26126267 PMC4493616

[17] Ramchurn SK, Goorah SS, Makhan M, Moheeput K. Excess mortality as an epidemic intelligence tool in chikungunya mapping. Euro Surveill. 2008;13(7). Epub 2008/05/01. . 18445418 10.2807/ese.13.07.08039-en

[18] Arpino C, Curatolo P, Rezza G. Chikungunya and the nervous system: what we do and do not know. Rev Med Virol. 2009;19(3):121–9. Epub 2009/03/11. 10.1002/rmv.606 . 19274635

[19] Gerardin P, Couderc T, Bintner M, Tournebize P, Renouil M, Lemant J, et al Chikungunya virus-associated encephalitis: A cohort study on La Reunion Island, 2005–2009. Neurology. 2016;86(1):94–102. 10.1212/WNL.0000000000002234 . 26609145

[20] Oehler E, Fournier E, Leparc-Goffart I, Larre P, Cubizolle S, Sookhareea C, et al Increase in cases of Guillain-Barre syndrome during a Chikungunya outbreak, French Polynesia, 2014 to 2015. Euro Surveill. 2015;20(48):30079 10.2807/1560-7917.ES.2015.20.48.30079 . 26690898

[21] Queyriaux B, Simon F, Grandadam M, Michel R, Tolou H, Boutin JP. Clinical burden of chikungunya virus infection. Lancet Infect Dis. 2008;8(1):2–3. 10.1016/S1473-3099(07)70294-3 . 18156079

[22] Sissoko D, Malvy D, Ezzedine K, Renault P, Moscetti F, Ledrans M, et al Post-epidemic Chikungunya disease on Reunion Island: course of rheumatic manifestations and associated factors over a 15-month period. PLoS Negl Trop Dis. 2009;3(3):e389 10.1371/journal.pntd.0000389 19274071 PMC2647734

[23] Essackjee K, Goorah S, Ramchurn SK, Cheeneebash J, Walker-Bone K. Prevalence of and risk factors for chronic arthralgia and rheumatoid-like polyarthritis more than 2 years after infection with chikungunya virus. Postgraduate Med J. 2013;89(1054):440–7. 10.1136/postgradmedj-2012-131477 . 23645664

[24] Moro ML, Grilli E, Corvetta A, Silvi G, Angelini R, Mascella F, et al Long-term chikungunya infection clinical manifestations after an outbreak in Italy: a prognostic cohort study. J Infect. 2012;65(2):165–72. 10.1016/j.jinf.2012.04.005 . 22522292

[25] Soumahoro MK, Boelle PY, Gauzere BA, Atsou K, Pelat C, Lambert B, et al The Chikungunya epidemic on La Reunion Island in 2005–2006: a cost-of-illness study. PLoS Negl Trop Dis. 2011;5(6):e1197 Epub 2011/06/23. 10.1371/journal.pntd.0001197 21695162 PMC3114750

[26] Shepard DS. Cost and burden of dengue and chikungunya from the Americas to Asia. Dengue Bulletin 2010; 34. WHO Regional Office for South-East Asia, 2010.

[27] Bloch D. The cost and burden of chikungunya in the Americas. New Haven EliScholar, USA: Yale University; 2016.

[28] Eckels KH, Harrison VR, Hetrick FM. Chikungunya virus vaccine prepared by Tween-ether extraction. Appl Microbiol. 1970;19(2):321–5. Epub 1970/02/01. 4985431 10.1128/am.19.2.321-325.1970 PMC376676

[29] Harrison VR, Eckels KH, Bartelloni PJ, Hampton C. Production and evaluation of a formalin-killed Chikungunya vaccine. J Immunol. 1971;107(3):643–7. Epub 1971/09/01. . 4999088

[30] Tiwari M, Parida M, Santhosh SR, Khan M, Dash PK, Rao PV. Assessment of immunogenic potential of Vero adapted formalin inactivated vaccine derived from novel ECSA genotype of Chikungunya virus. Vaccine. 2009;27(18):2513–22. 10.1016/j.vaccine.2009.02.062 . 19368794

[31] Kumar M, Sudeep AB, Arankalle VA. Evaluation of recombinant E2 protein-based and whole-virus inactivated candidate vaccines against chikungunya virus. Vaccine. 2012;30(43):6142–9. 10.1016/j.vaccine.2012.07.072 . 22884660

[32] Levitt NH, Ramsburg HH, Hasty SE, Repik PM, Cole FE, Lupton HW. Development of an attenuated strain of chikungunya virus for use in vaccine production. Vaccine. 1986;4(3):157–62. 3020820 10.1016/0264-410x(86)90003-4

[33] Edelman R, Tacket CO, Wasserman SS, Bodison SA, Perry JG, Mangiafico JA. Phase II safety and immunogenicity study of live chikungunya virus vaccine TSI-GSD-218. Am J Trop Med Hyg. 2000;62(6):681–5. . 11304054 10.4269/ajtmh.2000.62.681

[34] Gorchakov R, Wang E, Leal G, Forrester NL, Plante K, Rossi SL, et al Attenuation of Chikungunya virus vaccine strain 181/clone 25 is determined by two amino acid substitutions in the E2 envelope glycoprotein. J Virol. 2012;86(11):6084–96. 10.1128/JVI.06449-11 22457519 PMC3372191

[35] McClain DJ, Pittman PR, Ramsburg HH, Nelson GO, Rossi CA, Mangiafico JA, et al Immunologic interference from sequential administration of live attenuated alphavirus vaccines. J Infect Dis. 1998;177(3):634–41. 9498442 10.1086/514240

[36] Weaver SC, Osorio JE, Livengood JA, Chen R, Stinchcomb DT. Chikungunya virus and prospects for a vaccine. Expert Rev Vaccines. 2012;11(9):1087–101. Epub 2012/11/16. 10.1586/erv.12.84 23151166 PMC3562718

[37] Powers AM. Chikungunya virus control: is a vaccine on the horizon? Lancet. 2014 Epub 2014/08/19. 10.1016/S0140-6736(14)61290-3 . 25132506 PMC6443378

[38] Hallengard D, Kakoulidou M, Lulla A, Kummerer BM, Johansson DX, Mutso M, et al Novel attenuated Chikungunya vaccine candidates elicit protective immunity in C57BL/6 mice. J Virol. 2014;88(5):2858–66. Epub 2013/12/29. 10.1128/JVI.03453-13 24371047 PMC3958085

[39] Chu H, Das SC, Fuchs JF, Suresh M, Weaver SC, Stinchcomb DT, et al Deciphering the protective role of adaptive immunity to CHIKV/IRES a novel candidate vaccine against Chikungunya in the A129 mouse model. Vaccine. 2013;31(33):3353–60. Epub 2013/06/04. 10.1016/j.vaccine.2013.05.059 23727003 PMC3731778

[40] Roy CJ, Adams AP, Wang E, Plante K, Gorchakov R, Seymour RL, et al Chikungunya vaccine candidate is highly attenuated and protects nonhuman primates against telemetrically monitored disease following a single dose. J Infect Dis. 2014;209(12):1891–9. Epub 2014/01/10. 10.1093/infdis/jiu014 24403555 PMC4038141

[41] Plante K, Wang E, Partidos CD, Weger J, Gorchakov R, Tsetsarkin K, et al Novel chikungunya vaccine candidate with an IRES-based attenuation and host range alteration mechanism. PLoS Pathog. 2011;7(7):e1002142 10.1371/journal.ppat.1002142 21829348 PMC3145802

[42] Plante KS, Rossi SL, Bergren NA, Seymour RL, Weaver SC. Extended Preclinical Safety, Efficacy and Stability Testing of a Live-attenuated Chikungunya Vaccine Candidate. PLoS Negl Trop Dis. 2015;9(9):e0004007 10.1371/journal.pntd.0004007 26340754 PMC4560411

[43] Langsjoen RM, Haller SL, Roy CJ, Vinet-Oliphant H, Bergren NA, Erasmus JH, et al Chikungunya Virus Strains Show Lineage-Specific Variations in Virulence and Cross-Protective Ability in Murine and Nonhuman Primate Models. MBio. 2018;9(2). 10.1128/mBio.02449-17 29511072 PMC5844994

[44] Ramsauer K, Schwameis M, Firbas C, Mullner M, Putnak RJ, Thomas SJ, et al Immunogenicity, safety, and tolerability of a recombinant measles-virus-based chikungunya vaccine: a randomised, double-blind, placebo-controlled, active-comparator, first-in-man trial. Lancet infect Dis. 2015 Epub 2015/03/06. 10.1016/S1473-3099(15)70043-5 . 25739878

[45] Nasar F, Palacios G, Gorchakov RV, Guzman H, Da Rosa AP, Savji N, et al Eilat virus, a unique alphavirus with host range restricted to insects by RNA replication. Proc Natl Acad Sci USA. 2012;109(36):14622–7. Epub 2012/08/22. 10.1073/pnas.1204787109 22908261 PMC3437828

[46] Nasar F, Gorchakov RV, Tesh RB, Weaver SC. Eilat virus host range restriction is present at multiple levels of the virus life cycle. J Virol. 2015;89(2):1404–18. Epub 2014/11/14. 10.1128/JVI.01856-14 25392227 PMC4300653

[47] Erasmus JH, Needham J, Raychaudhuri S, Diamond MS, Beasley DW, Morkowski S, et al Utilization of an Eilat Virus-Based Chimera for Serological Detection of Chikungunya Infection. PLoS Negl Trop Dis. 2015;9(10):e0004119 10.1371/journal.pntd.0004119 26492074 PMC4619601

[48] Erasmus JH, Auguste AJ, Kaelber JT, Luo H, Rossi SL, Fenton K, et al A chikungunya fever vaccine utilizing an insect-specific virus platform. Nat Med. 2017;23(2):192–9. 10.1038/nm.4253 27991917 PMC5296253

[49] Akahata W, Yang ZY, Andersen H, Sun S, Holdaway HA, Kong WP, et al A virus-like particle vaccine for epidemic Chikungunya virus protects nonhuman primates against infection. Nat Med. 2010;16(3):334–8. 10.1038/nm.2105 20111039 PMC2834826

[50] Chang LJ, Dowd KA, Mendoza FH, Saunders JG, Sitar S, Plummer SH, et al Safety and tolerability of chikungunya virus-like particle vaccine in healthy adults: a phase 1 dose-escalation trial. Lancet. 2014 Epub 2014/08/19. 10.1016/S0140-6736(14)61185-5 . 25132507

[51] Smalley C, Erasmus JH, Chesson CB, Beasley DWC. Status of research and development of vaccines for chikungunya. Vaccine. 2016;34(26):2976–81. 10.1016/j.vaccine.2016.03.076 . 27026149

[52] Ahola T, Couderc T, Ng LF, Hallengard D, Powers A, Lecuit M, et al Therapeutics and vaccines against chikungunya virus. Vector Borne Zoonotic Dis. 2015;15(4):250–7. 10.1089/vbz.2014.1681 . 25897811

[53] Singh P, Chhabra M, Mittai V, Sharma P, Rizvi MA, Chauhan LS, et al Current research and clinical trials for a vaccine against chikungunya virus. Vaccine Development and Therapy. 2013;3:35–46.

[54] Whitehead SS, Subbarao K. Which Dengue Vaccine Approach Is the Most Promising, and Should We Be Concerned about Enhanced Disease after Vaccination? The Risks of Incomplete Immunity to Dengue Virus Revealed by Vaccination. Cold Spring Harb Perspect Biol. 2018;10(6). 10.1101/cshperspect.a028811 . 28716894 PMC5983189

[55] Henao-Restrepo AM, Longini IM, Egger M, Dean NE, Edmunds WJ, Camacho A, et al Efficacy and effectiveness of an rVSV-vectored vaccine expressing Ebola surface glycoprotein: interim results from the Guinea ring vaccination cluster-randomised trial. Lancet. 2015;386(9996):857–66. 10.1016/S0140-6736(15)61117-5 . 26248676

[56] Capeding MR, Chua MN, Hadinegoro SR, Hussain II, Nallusamy R, Pitisuttithum P, et al Dengue and other common causes of acute febrile illness in Asia: an active surveillance study in children. PLoS Negl Trop Dis. 2013;7(7):e2331 10.1371/journal.pntd.0002331 23936565 PMC3723539

[57] CFR-Code of Federal Regulations Title 21, Vol. 5. Revised as of April 21. Cite: 21CFR214. 2014.

[58] Mallilankaraman K, Shedlock DJ, Bao H, Kawalekar OU, Fagone P, Ramanathan AA, et al A DNA vaccine against chikungunya virus is protective in mice and induces neutralizing antibodies in mice and nonhuman primates. PLoS Negl Trop Dis. 2011;5(1):e928 10.1371/journal.pntd.0000928 21264351 PMC3019110

[59] Andre FE. How the research-based industry approaches vaccine development and establishes priorities. Dev Biol (Basel). 2002;110:25–9. . 12477303

[60] Rezza G. Do we need a vaccine against chikungunya? Pathog Glob Health. 2015;109(4):170–3. 10.1179/2047773215Y.0000000017 25971340 PMC4530554

[61] Rezza G. Vaccines against chikungunya, Zika and other emerging Aedes mosquito-borne viruses: unblocking existing bottlenecks. Future Virol. 2016;11:723–30.

[62] Wong G, Qiu X. Funding vaccines for emerging infectious diseases. Hum Vaccin Immunother. 2018;14(7):1760–2. 10.1080/21645515.2017.1412024 . 29194012 PMC6067896

[63] Brandler S, Ruffie C, Combredet C, Brault JB, Najburg V, Prevost MC, et al A recombinant measles vaccine expressing chikungunya virus-like particles is strongly immunogenic and protects mice from lethal challenge with chikungunya virus. Vaccine. 2013;31(36):3718–25. Epub 2013/06/08. 10.1016/j.vaccine.2013.05.086 . 23742993

[64] Hallengard D, Lum FM, Kummerer BM, Lulla A, Lulla V, Garcia-Arriaza J, et al Prime-boost immunization strategies against Chikungunya virus. J Virol. 2014;88(22):13333–43. 10.1128/JVI.01926-14 25210177 PMC4249109

[65] Chikungunya: Villepin refuse la polémique. Le Figaro. 15 October 2007. Available from: http://www.lefigaro.fr/sciences/2006/02/24/01008-20060224ARTWWW91671-nouveau_bilan_cas.php. [cited 21 December 2018].

